# Effectiveness of communication campaign on iron deficiency anemia in Kyzyl-Orda region, Kazakhstan: a pilot study

**DOI:** 10.1186/1471-2326-10-2

**Published:** 2010-03-17

**Authors:** Ainur Baizhumanova, Akio Nishimura, Katsuki Ito, Junichi Sakamoto, Nailya Karsybekova, Igor Tsoi, Nobuyuki Hamajima

**Affiliations:** 1 Department of Young Leaders' Program for Medical Administration and Politics, Graduate School of Medicine, Nagoya University, 65 Tsurumai-Cho, Nagoya, 466-8550, Japan; 2 Department of Education Training, Technology and Development, National Institute of Public Health, 2-3-6 Minami, Wako, 351-0197, Japan; 3 Department of Alimentary Dependent Diseases, Institute of Nutrition Issues, 49 Beibitshilik Str, Astana, 010000, Kazakhstan; 4 Kazakh Academy of Nutrition, National Academy of Sciences, 66 Klochkov Str, Almaty, 050000, Kazakhstan; 5 Department of Preventive Medicine/Biostatistics and Medical Decision Making, Graduate School of Medicine, Nagoya University, 65 Tsurumai-Cho, Nagoya, 466-8550, Japan

## Abstract

**Background:**

In 2004, wheat flour fortification (WFF) with iron was implemented in Kazakhstan as a public health strategy to increase the iron intake of all women of childbearing age and of children. In 2003, before starting the flour fortification program, a communication campaign on health education took place in a region with a high prevalence of iron deficiency anemia (IDA). The present study aimed to evaluate the prevalence of anemia, iron deficiency and IDA before and after the campaign. In addition, knowledge about IDA and its prevention, as well as awareness about fortified wheat flour, was assessed.

**Methods:**

The subjects of the study were women aged 15-49 years and children aged 2-14 years. The study was carried out in urban and rural areas of Kyzyl-Orda region in 2003 before (March) and after (December) the campaign. Blood samples were collected in order to measure hemoglobin and serum ferritin. In March 80 women and 57 children in the urban area, and 41 women and 41 children in the rural area, participated in the IDA testing. The corresponding participants in December numbered 62, 52, 52, and 57, respectively. The impacts of the communications and information received by participants during the campaign was surveyed with a questionnaire for 195 women in March and 198 women in December including some who participated in the IDA testing.

**Results:**

In March, the prevalence of anemia was 52.0% among 121 women and 58.1% among 98 children, and those with low iron reserve were 63.6%, 49.1% and IDA 40.5%, 11.0%, respectively. In December, the prevalence of anemia had significantly decreased among rural women (from 65.9% to 48.0%, p < 0.05) and among urban children (from 63.1% to 11.5%, p < 0.001). The prevalence of iron deficiency was significantly reduced among the children (from 51.1% to 24.8%, p < 0.001). IDA prevalence was meaningfully decreased among women in urban and combined areas (from 37.5% to 15.0% and 40.5 to 14.8%, respectively, p < 0.001) and among urban children (from 7.1% to 2.1%, p < 0.05). The surveys found that most women knew about IDA and its prevention and that the numbers were similar both in March and in December. The knowledge of the anti-anemic effect of wheat fortified flour improved significantly over the period of the campaign among women both in urban (from 48.5% to 80.9%, p < 0.001) and rural (from 69.8% to 88.6%, p < 0.001) areas.

**Conclusion:**

The study demonstrated that the communication campaign before implementation of WFF program was effectively carried out, giving a biological impact on hematological indices.

## Background

Iron deficiency anemia (IDA) is the most common and widespread nutritional disorder in Kazakhstan [1-11]. In 2004, as one of the comprehensive public health measures to achieve improvement in the country, a wheat flour fortification (WFF) program took place. Before implementation of the WFF, a communication campaign to build public awareness about IDA and its prevention, as well as about new product fortified wheat flour, was conducted. This campaign consisted of health education through different types of media, social mobilization, and marketing by civil society. The study aimed to evaluate the prevalence of anemia, iron deficiency and IDA before and after the campaign. In addition, improvement of awareness about IDA, its prevention, and fortified wheat flour were assessed.

## Methods

### Study location and population

Kyzyl-Orda region is situated in southern Kazakhstan, extending along both banks of the lower Syrdarya River. The Aral Karakum and Kyzylkum deserts, making it one of the hottest and most arid regions of Kazakhstan, cover a large part of this region. The total population of the region was about 603.800 in 2003, or about 4 percent of the country's population, and two-thirds of the population was urban. In that year, the region ranked lowest in per capita income, with industrial production representing about 1.1 percent of the country's total production. The principal industry is agriculture, specifically the breeding of cattle, growing rice and fishery. In addition to some social and economic difficulties, the region suffers from environmental pollution, a difficult climate, and high levels of pesticide concentration in the environment and salts of heavy metals. All of these factors negatively affect the health condition of the population. One of the most serious public health problems in the Kyzyl-Orda region is anemia. According to numerous studies the prevalence rate of anemia is the highest in the country [1-11]. Although many causes of anemia have been identified, such as hemoglobinopathies, parasitic infestation, chronic bleeding, and deficiencies of folic acid and vitamin C, evidence suggests that the majority of cases of anemia in Kazakhstan are due to negative iron balance [5].

### Study subjects

Kyzyl-Orda was chosen as a pilot region for the present study because the IDA prevalence in this region is the highest in the country. One city and one district were selected from six districts of the pilot region, which allowed for comparison of the differences in efficiency between urban and rural areas. The study consisted of two surveys with a time interval of 9 months. The first survey was conducted in March before the campaign and the second survey in December of the same year after the campaign.

A two-stage sampling process was used to select women for the study. In the first stage, Kyzyl-Orda city and Kazalinsk district were chosen because of the highest prevalence of IDA in the Kyzyl-Orda region. In the second stage, every sixth household was selected from the complete list of households registered at the selected health centres. Participants were selected by systematic random sampling. An initial household was selected at random and every sixth household was chosen from the list of households. The sampling frame was assumed to be complete since all women and children in Kyzyl-Orda region were required to be registered at their local health centre. Women aged 15-49 years and their children aged 2-14 years were invited to the clinics on designated days. The same selection method was used for both urban and rural areas as well as for March and December surveys.

The Ethical Committee of the Kazakh Academy of Nutrition and Ministry of Health of Kazakhstan approved the study. Prior to participating in the IDA testing, each woman was asked to sign a consent form giving permission for the collection of a blood droplet from her and her children.

From 200 households 200 women and 100 children were selected for our study. Of these, 121 women and their children were selected for IDA testing and interview. The remaining 79 women were selected only for the interview. In March, of those eligible women and children selected, approximately 60.5% of women and 98% of children participated in the IDA testing. Ninety seven percent of women attended the interview and that number included the women who participated in the IDA testing. In December the corresponding participation rates were 57%, 100% and 99% respectively. The main reasons of absence of women who did not participate in the IDA testing were: not at home, and/or not available to come to the clinic. Percentages of refusals among women in March and December were 1.0 and 0.5, correspondingly. Among those women who participated in IDA testing and interview 68 were able to participate in both surveys.

### Data collection

To evaluate the influence of the communication campaign participating women were interviewed by trained nurses at local health centres. The questionnaire included such topics as awareness on the health problems due to IDA, diets to prevent IDA and knowledge of healthy nutrition and fortified wheat flour.

The nurses also took blood samples from the participants. Hemoglobin (Hb) concentration of the capillary blood of the second drop of blood, being obtained by a puncture of a finger tip, using the HemoCue (HemoCue AB, Angelholm, Sweden) portable spectrophotometer. The venous blood sample was centrifuged and the separated serum was transported to the measure centre in frozen state, and serum ferritin (SF) was measured by the radio immune assay (Diagnostic Products Corporation, Los Angeles, CA) method. To define anemia and iron deficiency we used WHO cut-off criteria for developing countries [12,13]. Table 1 shows the definitions of anemia according to age group, applied for this study. The assessment of iron deficiency was performed through a measurement of SF. Cut off points for low level of ferritin or iron depletion was <12 μg/L for children under 5 years and <15 μg/L for children ≥ 5 years of age and women. IDA was defined in this study as those with anemia described in Table 1 and SF < 12 or 15 μg/L depend on age. Since 68 women participated in both surveys, their data were linked and the differences between the two surveys were compared.

**Table 1:** Definition of anemia according to blood hemoglobin (Hb) concentration (g/dl)

Subjects	Anemia			
	
	Severe anemia	Moderate anemia	Mild anemia	No anemia
**Children**				
6-59 months	<7	7-9.9	10-10.9	≥11
5-11 years	<7	7-9.9	10-11.4	≥11.5
12-14 years	<7	7-9.9	10-11.9	≥12

**Women**				
15-49 years	<7	7-9.9	10-11.9	≥12

### Statistical Analysis

Mean ± standard deviations were calculated for Hb and SF. The means of Hb and SF were examined with a paired t-test. Chi-square test and Fisher's exact test (in case of subjects <10) were also used to compare the distributions of categorical data. Two sided P-values < 0.05 were considered as significant. Statistical analyses were conducted using SPSS for Windows Version 11.5 (SPSS, Chicago, IL, USA).

## Results

### Hematological indices

Table 2 shows the percentages of subjects with anemia. Significant improvement in the prevalence of anemia between two surveys was observed for the women in the rural area (p < 0.05), and the children in the urban area (p < 0.001). The improvement in the prevalence of anemia was significant (p < 0.001) for the children in urban area. The data shows that it was also significant in the urban and rural areas combined. The prevalence of iron deficiency and IDA is shown in Table 3. The iron deficiency prevalence was significantly reduced among the children in urban, rural, and combined areas. The prevalence of IDA was significantly decreased among women in urban, urban and rural combined areas and urban children.

**Table 2:** Percentages and their 95% confidence intervals (CI) of subjects with anemia in the first (March) and second (December) surveys according to survey area

Women						
**Area**	**Date**	**N**	**Percentage and 95% CI of subjects with anemia**			
			
			**Mild**	**Moderate**	**Severe**	**Total**
			
Urban	March	80	30.0 (20.0, 40.0)	13.7 (6.2, 21.2)	1.2 (-1.2, 3.6)	45.0 (34.1, 55.9)
	December	62	33.9 (22.1, 45.7)	8.1 (1.3, 14.9)	1.6 (-1.5, 4.7)	43.6 (31.3, 55.9)
Rural	March	41	29.3 (15.4, 43.2)	29.3 (15.4, 43.2)	7.3 (-0.7, 15.3)	65.9 (51.4, 80.4)
	December	52	28.8 (16.5, 41.1)	19.2 (8.5, 29.9)	0.0	48.0 (34.4, 61.6)*
Total	March	121	29.7 (21.6, 37.8)	19.0 (12.0, 26.0)	3.3 (0.1, 6.5)	52.0 (43.1, 60.9)
	December	114	31.6 (23.1, 40.1)	13.1 (6.9, 19.3)	0.9 (-0.8, 2.6)	45.6 (36.5, 54.7)

**Children**						

Urban	March	57	49.1 (36.1, 62.1)	12.3 (3.8, 20.8)	1.7 (-1.7, 5.1)	63.1 (50.6, 75.6)
	December	52	11.5 (2.8, 20.2)	0.0	0.0	11.5 (2.8, 20.2)**
Rural	March	41	34.1 (19.6, 48.6)	19.5 (7.4, 31.6)	0.0	53.6 (38.3, 68.9)
	December	57	49.1 (36.1, 62.1)	3.5 (-1.3, 8.3)	0.0	52.6 (39.6, 65.6)
Total	March	98	42.8 (33.0, 52.6)	15.3 (8.2, 22.4)	1.0 (-1.0, 3.0)	58.1 (48.3, 67.9)
	December	109	31.2 (22.5, 39.9)	1.8 (-0.7, 4.3)	0.0	33.0(24.2, 41.8)**

* p < 0.05, ** p < 0.001 by a chi-square test for the difference in the percentage between March and December

**Table 3:** Percentage and 95% CI of women and children with iron deficiency and iron deficiency anemia in the first (March) and second (December) surveys in urban and rural areas

Women				
**Area**	**Date**	**N**	**Percentage and 95% CI**	
			
			**Iron deficiency**	**Iron deficiency anemia**

Urban	March	80	66.2 (55.8, 76.6)	37.5 (26.9, 48.1)
	December	60	45.0 (32.4, 57.6)	15.0 (6.0, 24.0)**
Rural	March	41	58.5 (43.4, 73.6)	24.0 (10.9, 37.1)
	December	48	54.2 (40.1, 68.3)	14.6 (4.6, 24.6)
Total	March	121	63.6 (55.0, 72.2)	40.5 (31.8, 49.2)
	December	108	49.1 (39.7, 58.5)	14.8 (8.1, 21.5)**

**Children**				

Urban	March	56	41.1 (28.2, 54.0)	7.1 (0.4, 13.8)
	December	48	20.8 (9.3, 32.3)**	2.1 (-2.0, 6.2)*
Rural	March	36	66.7 (51.3, 82.1)	16.7 (4.5, 28.9)
	December	57	28.1 (16.4, 39.8)**	14.0 (5.0, 23.0)
Total	March	92	51.1 (40.9, 61.3)	11.0 (4.6, 17.4)
	December	105	24.8 (16.5, 33.1)**	8.6 (3.2, 14.0)

*p < 0.05, **p < 0.001 by a chi-square test for the difference in the percentage between March and December

**Figure 1:**
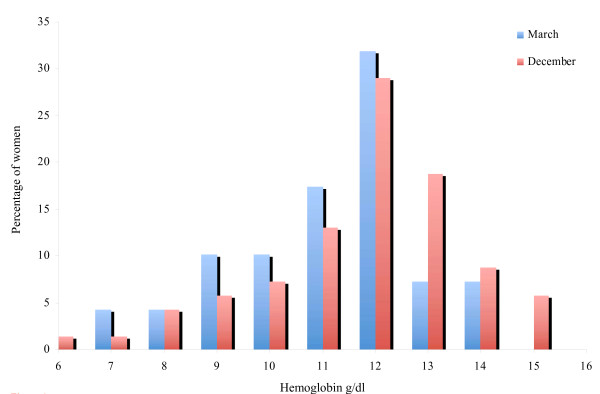
**Distribution of blood hemoglobin for women aged 15-49 years before (March, N = 121) and after (December, N = 114) the campaign**.

Table 4 presents the average (Hb), geometric mean (SF) and standard deviation among 68 women who participated in both surveys. While the improvement in average Hb was significant among rural women, it was not significant among urban women. On the contrary, geometric mean of SF was significantly improved among the urban women. Figure 1 shows Hb distribution for all women before (March) and after (December) the campaign, the distribution for the survey on December was slightly shifted to the left compared with that of March.

**Table 4:** Hemoglobin (Hb) and serum ferritin (SF) levels of women in both surveys in urban and rural areas

	Area	N	March		December	
				
			Average	± SD	Average	± SD
**Hb**, g/dL						
	Urban	44	11.9	± 3.6	12.2	± 1.9
	Rural	24	10.7	± 2.1	11.8*	± 1.9
	Total	68	11.5	± 3.2	12.0	± 1.9

**SF**, μg/L						
	Urban	43	7.6	± 10.3	14.2**	± 21.9
	Rural	23	8.0	± 11.7	9.7	± 9.9
	Total	66	7.7	± 10.7	12.4**	± 18.9

SF data presented as geometric means

*p < 0.05, **p < 0.001 by a paired t-test

### Questionnaire survey

**Figure 2:**
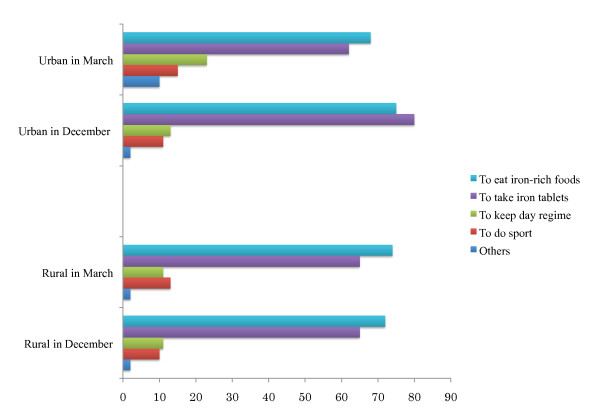
**Responses to the question, "How to prevent iron deficiency anemia?" (%)**. * Day regime doesn't include diet.

**Figure 3:**
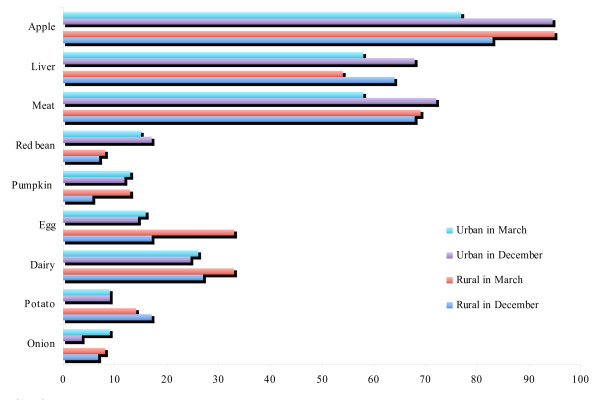
**Percentage of subjects who selected the item as iron-rich product for the question, "What products contain iron?"**. * Apple, liver, and meat are iron-rich foods.

As shown in Table 5, even in the first survey almost all of the women had heard about IDA. Among them more than 60.0% of both rural and urban women have chosen to eat iron rich foods and to take iron tablets as prevention against IDA. About 20.0% of women have chosen such answers as to go to sport and keep day regime (Figure 2). In the question about products rich in iron, the majority of women chose apple, meat, and liver, which are truly iron-rich products. Some of them chose eggs, potato, pumpkin and onion that contain less bioavailable iron or small amount of iron; the other of them chose dairy products and red beans that are even inhibiting iron absorption (Figure 3). About the knowledge on fortified wheat flour, a significant improvement was observed both among urban and rural women (Table 5).

**Table 5:** Percentage and 95% CI of women who answered, "yes" to the questions listed on the footnote

	Percentage and 95% CI			
	
	Urban		Rural	
		
	March	December	March	December
	N = 132	N = 110	N = 63	N = 88
	
Q1	96.2 (93.0, 99.5)	100.0 (100.0)	98.4 (95.3, 101.5)	99.0 (97.0, 101.1)
**Q2**	48.5 (40.0, 57.0)	80.9 (73.5, 88.2)**	69.8 (58.5, 81.1)	88.6 (82.0, 95.2) *

Q1: "Did you hear about iron deficiency anemia?"

Q2: "Do you know that the flour can be enriched by iron on manufacture?"

*p < 0.05, **p < 0.01 by a chi-square test

The sources of information on IDA were assessed in two questions: generally (Table 6) and specifically through visual tools (Table 7). More significant sources of information were medical workers, TV, radio, and special brochures among urban women, and special brochures and medical posters among rural women (Table 6). Medical workers were marginally significant sources of information among rural women (p < 0.09). As shown in Table 7, the roles of TV, medical posters, posters in shops, and trademarks became significantly larger in the urban area, and TV and medical posters did in the rural area.

**Table 6:** Sources of information about iron deficiency anemia

	Urban %		Rural %	
		
	March	December	March	December
	N = 132	N = 110	N = 63	N = 88
Friend, colleague	9 (6.8)	8 (7.3)	5 (7.9)	4 (4.5)
Family member	20 (15.2)	13 (11.8)	5 (7.9)	2 (2.3)
School	11 (8.3)	7 (6.4)	3 (4.8)	6 (6.8)
Medical worker	109(82.6)	104 (94.5)*	54 (85.7)	83 (94.3) †
TV	31 (23.5)	59 (53.6)**	9 (14.3)	18 (20.5)
Newspaper	31 (23.5)	21 (19.1)	4 (6.3)	12 (13.6)
Radio	13 (9.8)	37 (33.6)**	2 (3.2)	7 (8.0)
Special brochure	32 (24.2)	16 (14.5)*	4 (6.3)	17 (19.3)*
Leaflet	5 (3.8)	8 (7.3)	3 (4.8)	5 (5.7)
Medical poster	40 (30.3)	42 (38.2)	6 (9.5)	25 (28.4)*

The sources of information were asked by the question: "Whence did you hear or receive the information about iron deficiency anemia in the past one year ?" *p < 0.05, **p < 0.001, †p < 0.1 by a chi-square test or Fisher's exact test in case that the respective 2 by 2 tables include a number <10

**Table 7:** Visually received information sources of two surveys of the study

	Urban %		Rural %	
		
	March	December	March	December
	N = 132	N = 110	N = 63	N = 88
Newspaper article	23 (17.4)	18 (16.4)	4 (6.3)	8 (9.1)
TV	50 (37.9)	80 (72.7) **	21 (33.3)	60 (68.2) **
Medical poster	51 (38.6)	77 (70.0) **	1 (39.7)	52 (59.1) *
Poster in the shop	4 (3.0)	10 (9.1) *	0 (0.0)	4 (4.5)
Trade mark of fortified	1 (0.8)	8 (7.3) *	0 (0.0)	3 (3.4)
wheat flour with iron				

The visually received information sources were asked by the question: "Where did you see the information about iron deficiency anemia in the past one year?"

*p < 0.05, **p < 0.001 by a chi-square test or Fisher's exact test in case that respective 2 by 2 tables include a number <10.

## Discussion

In March, the prevalence of anemia was high (52.0% among the women and 58.1% among the children), and those with iron deficiency and IDA were prevalent (63.6%, 51.1% for iron deficiency and 40.5%, 11.0% for IDA, respectively). In December the prevalence of anemia was significantly reduced from 65.9% to 48.0% among rural women, and from 63.1% to 11.5% among urban children. The dramatic change in Hb status of urban children and the slight improvement without any behavioral change in rural women can be explained by intake of variety of fruits and vegetables taken during summer and autumn seasons.

The percentage for the iron deficiency among the children was significantly reduced from 51.1% to 24.8%. Significant improvement on IDA prevalence and some improvement in iron deficiency prevalence were observed among women in urban and urban and rural combined areas that can most possibly be due to taking iron pills after education campaign. Although the effects of the campaign on IDA were not consistent across the subgroups, they were unexpectedly large. The surveys found that most of women knew about IDA and that these numbers did not differ significantly in March and December. However, the prevalence of anemia, iron deficiency and IDA remained high among women. The main reasons for that are irrational nourishment, a strong tradition of drinking tea (high content of tannins in the tea) during meals, high birth rates, low levels of education, leading to lack of a culture of nourishment and habits of healthy nutrition [4,5]. These factors are not susceptible to change over a short time period.

Knowledge about fortified wheat flour among the women improved significantly both in urban (from 48.5% to 80.9%) and rural (from 69.8% to 88.6%) areas.

Food fortification is one of the most cost-effective and sustainable strategies for increasing iron intake in general populations [14-16]. Several trials have found that the consumption of iron-fortified staple foods or condiments improves iron status. The fortified products were iron-fortified fish sauce [17], salt fortified with microencapsulated iron, iodine, and vitamin A [18], iron-fortified curry powder [19], iron-fortified soy sauce [20], iron-fortified milk and drinking water [21], and sugar fortified with vitamin A and iron [22]. Forty-eight countries are currently enacting fortification programmes through voluntary or mandatory legislation. Twenty-eight of these countries fortify with iron and folic acid. Iron fortified flours have been used in different countries of the world including China, Venezuela and other American countries [23-26]. In Kazakhstan flour of the highest and first types was selected as the quality food vehicle, since it practically was present on each meal table, independent of family income. National food is based on the use of these types of flour. With the grinding of wheat flour almost 90% of all micronutrients are lost. The enrichment of wheat flour makes it possible to restore that loss.

Concerning the effects of the communication campaign on public awareness about the problems of anemia and importance of its prevention using fortified foods, however, there was limited information on the effectiveness. This study added findings on the effects of the campaign.

Several drawbacks of this study should be pointed out. Since the two surveys of the present study were made in March and December, that could influence the improvement on anemia and IDA prevalence and iron storage. We assume that March was after the period when the vegetables and fruits were relatively expensive, while December was after the period when vegetables and fruits were cheap and available. Secondly, whilst the effects on Hb and SF were examined with the subjects sampled before and after the campaign, not all of the subjects were the same for the March and December studies, so that the evidence produced must be considered indirect. However, improvement was observed among the 68 women who participated in both studies. Thirdly, SF might not reflect iron storage precisely. SF is an acute-phase reactant protein, which is elevated in response to infection. The underlying infectious diseases could mask the iron shortage among superficially healthy subjects [27]. However in our study we used the SF level indicator because its determination was widely used as a test to diagnose IDA. Iron stores decreases before serum iron levels, erythrocytes and Hb level decrease in the course of IDA. SF level is the best indicator for the women and children iron storage status and it is more sensitive and reliable than the other parameters.

Our results indicated unexplained inconsistency in Hb and SF levels between urban and rural women. Most likely this inconsistency is related to differential food access and approaches used by subjects to prevent IDA. Although health education had an influence on reduction of IDA and increases in iron stores in women, the positive effect on iron status will be temporary if these women's diets do not contain adequate bioavailable iron. And in this regard, WFF implementation will be very timely and helpful for sustainable improvement on anemia in the pilot region.

## Conclusion

The study demonstrated that the communication campaign was effectively carried out in Kazakhstan before implementation of the WFF program, giving a biological impact on hematological indices.

## Competing interests

The authors declare that they have no competing interests.

## Authors' contributions

All authors contributed to study design. HN, NA, IK, and JS have been involved in drafting the manuscript, revising it critically for important intellectual content, and have given final approval of the version to be published. NA conceived of the study, performed statistical analysis, and interpreted analyzed data. NK and TI coordinated the study, and contributed to acquisition of data. BA is principal author of the paper, had full access to all data, and is guarantor. All authors contributed to manuscript drafting and revision and approved the final manuscript.

## Pre-publication history

The pre-publication history for this paper can be accessed here:

http://www.biomedcentral.com/1471-2326/10/2/prepub
